# Imprecise Data and Their Impact on Translational Research in Medicine

**DOI:** 10.3389/fmed.2020.00082

**Published:** 2020-03-19

**Authors:** Enrico Capobianco

**Affiliations:** Institute of Data Science and Computing, University of Miami, Miami, FL, United States

**Keywords:** big data, electronic health records, translational science, clinical decision support systems, anticipative adaptive inference

## Abstract

The medical field expects from big data essentially two main results: the ability to build predictive models and the possibility of applying them to obtain accurate patient risk profiles and/or health trajectories. Note that the paradigm of precision has determined that similar challenges need to be faced in both population and individualized studies, namely the need of assembling, integrating, modeling, and interpreting data from a variety of information sources and scales potentially influencing disease from onset to progression. In many cases, data require computational treatment through solutions for otherwise intractable problems. However, as precision medicine remains subject to a substantial amount of data imprecision and lack of translational impact, a revision of methodological inference approaches is needed. Both the relevance and the usefulness of such revision crucially deal with the assimilation of data features dynamically interconnected.

## Introduction

Recently, Hulsen et al. (1) describedthe role of Big Data in shaping opportunities and challenges in current biomedical research. The key synergy between big data analytics and hypothesis-driven methods points out their role of mutually influencing drivers of change in clinical practice. Similarly, Capobianco (2) illustrated Big Data integrative vs. evidence-based medicine approaches in oncology, identifying synergy between two foundational PM principles, individualization (especially of treatment), and systems inference (relying on data connectivity). Quite evidently, the two addressed synergies run in parallel.

The literature on such topics includes focus on general issues, for example public health (3) or health economics (4), and more specific aspects such as data harmonization, semantic enrichment, and data science challenges (5) or redefinition of the meaning of “normality” in view of novel patient stratifications (6, 7), just to mention a few among the many studies contributing with general reviews and perspectives.

The scope of the present article is to contextualize further these concepts by leveraging various data dimensions in relation to unmet needs in the clinical practice.

## Big Data Gold Standard? Not Yet…

Precision and Translational Medicine require integration and analysis of large datasets with genetic, lifestyle, environmental, biochemical, imaging, clinical information, all possibly matched together. The result of such fusion should reveal high-resolution patient pictures, such as specific individual conditions determining the pathophysiological state at a certain time or over an interval. Importantly, the more detailed and accurate the information flow through time is and the more the monitored patient risk profiles and health trajectories can provide useful disease scenarios and related prognostic paths.

Consider for instance complex diseases, where it is well-known the complexity of defining patient's prognosis. The difficulty of establishing risk factors, the importance of predicting how patient conditions progress, the role played by comorbidities, these are just examples of problems that would require the support of *data performance indicators* as critical part of a data-centric approach. The indicators would need to assess: (a) Diagnostic accuracy, and thus improve in-depth disease characterization; (b) Earlier intervention, to enable a preventive use of data; (c) Targeted treatment, to exploit knowledge from multi-sourced cross-talking data types; (d) Increased drug efficacy, with a role for repurposing and repositioning all the knowledge in the current warehouse resources.

Although the wealth of available data should in principle facilitate the process of quantifying a series of impacts at both individual and population levels, there are *bottlenecks* of a various nature that complicate the evaluation of data potential and effects. Apart from the uncertainty due to noisy observations, there is often a substantial ambiguity in evaluations based on empirical data and imprecise records. Structural limitations arise not only from the data, but also from the medical practice. It is necessary to point out two evident factors: (1) the still limited use of clinically actionable data in practice, considering that *clinical trials* are mandatory for an evaluation of safety and efficacy of novel therapies, (2) the fact that results from *randomized clinical trials* are hard to extrapolate to daily clinical practice due to patient heterogeneity.

## Revised Approach: Act Early

Beyond these described bottlenecks, the importance of prevention in individualized medicine has increased determining centrality for inference of a wide spectrum of big data types and inducing the need of revising the methodological approach to leverage *anticipative adaptive inference*. This approach reflects the major improvements achieved in detecting early disease signs, marks or symptoms with wearable, mobile, sensor technologies exploiting the dynamic nature of many factors informing on patient health status. In turn, advantages were gained in domains such as: (a) Timely choice of treatments to lower mortality and increase cost-effective cures (b) Effective risk assessment in relation to disease variants (c) Identification of robust prognostic indicators (i.e., relevant at the time of diagnosis) (d) Construction of best informed patient trajectories (via continuous monitoring).

Among the major problems that remain, two can be summarized as follows: (1) Integration of heterogeneous information from different sources, which implies need of harmonization before proper assessment of their synergistic predictive potential (2) Extraction of clinical value from methods leveraging structured and unstructured data features. Ultimately, the validity of big data depends on their degree of *accuracy*. This for example impacts the formulation of diagnoses for a variety of cases. Clearly enough, patients present inherent variability that complicates the possibility of explaining or validating which factors differentially contribute to disease characterization. Such factors may be considered and measured to a variable precision level, and their inclusion in well-designed models would represent the real added value that Big Data may offer in disease knowledge by increasing the interpretability of diverse but potentially correlated information layers.

Complex diseases are highly heterogeneous dynamic processes whose causes, course of evolution, treatment and response to treatment determine a multitude of possible patient health trajectories. Therefore, the precision which is required crucially matches the need of early interventions, ideally at the stage of identification of specific molecular patterns. Often, treatment at early stages leads to substantial reduced risk of disease progression, and development of molecular and clinical biomarkers at early disease stages is associated with optimal chances of success from intervention. In summary, it is critical to anticipate disease onset identification by shortening the disease trajectory length measured by the temporal window between molecular trigger and clinical intervention.

**Figure 1:**
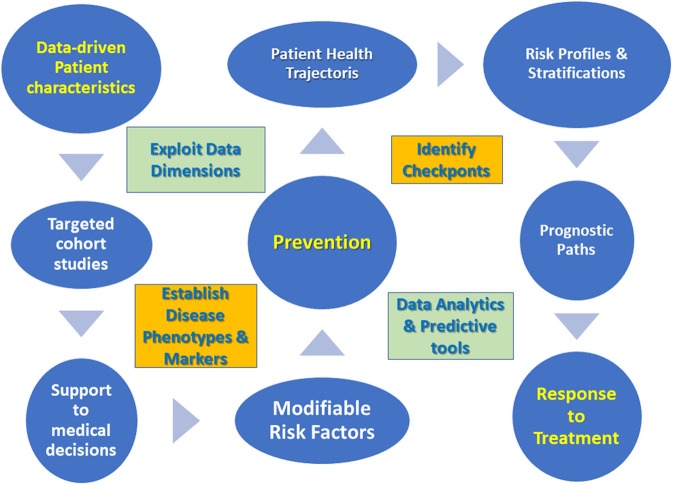
Data management guidelines for translational medicine approach.

Critically enough the challenges of daily clinical practice impose a few guidelines when it comes to data management [see also (8) for an innovation management perspective], then summarized in Figure 1.

➢ Focus on patient characteristics emerging from new data dimensions.
➢ Perform targeted cohort studies based on established disease phenotypes and markers.
➢ Develop support to medical decisions via data analytics tools.
➢ Determine modifiable risk factors to improve prevention in pre-disease cohorts.
➢ Characterize trajectories of disease progression or patient's health status by identifying checkpoints.
➢ Draw patient prognostic paths from risk profiles, stratifications and trajectory clusters.
➢ Develop predictive tools centered on response to treatment.

## Translational Potential

A recent study (9) has indicated a number of macrolevels involved in the process of building a *clinical decision support system* (CDSS), and they are: (1) *Data* (intended as lab work with a variety of specified thresholds, derived measurements, diagnostic codes etc.) (2) *Algorithms*, including methods chosen to handle missing data and construct variables from raw data (i.e., features), and (3) *Decision support* (model output interpretation, autonomy, usefulness). Once contextualized, these three macrolevels must be considered in temporal terms for a best possible assessment, especially regarding how to handle the variation components identified by the inherent (biological) variability and the so-called algorithmic drift (not induced by human intervention).

About decision making processes, a general definition of clinical decision support systems is currently lacking. Even if the reference goes to Kawamoto et al. (10), the reason of the difficulty in defining CDSS relies in their nature of sociotechnical systems (11). Among the various CDSS characteristics, two are especially seen as necessary: (a) Leverage of disease knowledge from which to formulate risk predictions (b) Integration between relevant stakeholders at hospital site. The relevance of building and integrating knowledge usable by CDSS implies prioritization in which one aspect is focal, how to assess the clinical usability of prediction models applicable through CDSS. This topic was considered in Kappen and Peelen (12) and Kappen et al. (13) by invoking a 3-step approach based on (1) Identifying the scope of using the predictive model (purpose, utility, target patients) (2) Establishing performance measures that refer to such scope (methodological aspects) (3) Assessing feasibility for clinical practice in view of available evidence more or less supportive of results (validation akin to applicability).

CDSS suggest to algorithmically treat problems characterized by high levels of imprecision, and this may indicate a potential role for soft computing and fuzzy logic methods, which may solve complexities too difficult to model at the mathematical level by leveraging the uncertainty [(14, 15) among other authors] and approximation [see the seminal work of (16)]. These methods can also be combined with probabilistic reasoning thus forming a relevant base for the field of approximate reasoning as a strategy to manage the imprecision of knowledge in inference tasks [see for instance (17)].

Quite clearly, *electronic health records* (EHR) represent a mine of information for CDSS, but without adequate consideration of context, EHR are a type of data that can lead to biased results. Considering their nature of a built-in dynamic process, EHR have value beyond observational databases and indirectly measure the patient's state from a variety of recordings. It is still largely underestimated the challenge of data integration in terms of augmented complexity. This is in part due to the volume of added features that a model must manage and in part is due to model design and structure, both to be adapted to an increased complexity.

For methodological and inferential purposes, *networks science* can be profitably used in combination with EHR (large-scale) and also in support of N-of-1 approaches (single-patient inference) to provide therapeutic guidelines based on the identification of drug combinations and the selection of relevant disease biomarkers (18). In parallel, three impact areas of data work have been proposed by Fiske et al. (19): one involves digital practices, another is centered on interpretation and contextualization to discriminate between information types, and one refers to inclusion and interaction for a more effective engagement of stakeholders [see also (20)].

Models of potential interest are those inspired by collaborative filtering and based on systems leveraging on reviews and built to recommend particular types of care provided to patients. Such models would thus enable centrality of patient-specific care and patient satisfaction by considering choices based on preferences and in consideration of context aspects and transparency principles [see for instance (21)].

## Data Analytics: Principled Development

A common way of thinking about big data analytics tools involves the design and construction of a scoring system able to assign value to a series of CDSS components leveraging data features and outcomes from methods application. These tools must obey a series of automated rules and algorithmic steps to perform predictions, which usually translates into estimating probabilities of disease relapse, defining risk profiles, drawing prognostic paths and health trajectories while leveraging the multilevel data characteristics to achieve satisfactory reproducibility and generalizability standards.

The automated data-driven processes underlying CDSS can likely enhance throughput applicability and facilitate the synergy with other healthcare process components, those necessary for instance to patient stratifications and based on multifactorial data or disease reclassification. It is well-known that alternative treatments exert differential effects on patients, something known as *heterogeneity of treatment effects* (HTE). In such regards, the challenges that the data embed refer substantially to (a) Structural aspects, i.e., observed vs. unobserved data features, and (b) Analytical aspects, i.e., what HTE features are predictive and what are not, also considering what is possible to estimate and what instead is destined to remain unknown.

*Data feature engineering* is considered central to useful interpretation in data science applications. In clinical research, expert knowledge can be especially relevant to the construction of feature sets applicable to predictive models. In contrast with such domain-centered approach, model-based feature engineering leverages on classic topics involving data dimensionality and heterogeneity in view of the type of learning approach (supervised or not) that would be required. A common strategy is establishing the relative importance of data features through suitably constructed scores for the purpose of assessing their predictive contribution to models. Even if limitations remain about the interaction between features (difficult to model and interpret) and the identification of influential or tipping points [see (22)], among the most feasible solutions that can address generalization there is the one of learning multiple models by creating ensembles (examples are bagging, stacking, boosting etc.) [see, among others (23)].

A recent theoretical framework has also been presented with the emphasis posed on three major principles: predictability, computability, and stability. They summarize, respectively, the compatibility of the model with the natural data generating processes, the algorithm efficiency, and the sensitivity referred to data and model perturbation for purposes of reproducibility and interpretability (24).

## Challenges Ahead

Given the expected high-resolution patient stratifications (25), what are good criteria to determine an optimal granularity, how this could be adapted to treatment specificity, EHR-rephenotyped diseases, patient geo-characteristics or multifaceted risk profiles?

Take cancer subtypes, for instance. In defining cancer subtypes, their molecular characterization is a criterion for treatment personalization treatment and selection into clinical trials. However, a Machine Learning tool *ad-hoc* for precision studied allowing translational applications is currently lacking. An effort in the direction of precision cancer therapy needs to address the knowledge improvement with regards to primary site of origin and accurate subtyping (in 2–5% of metastatic cancer patients cannot be located the primary site, thus left with a classified cancer of unknown primary and poor prognosis to only empiric treatment and insurgence of comorbidities). Flynn et al. (26) built pan-cancer classifiers to predict multiple cancer primary site of origin from metastatic tumor samples.

Manrai et al. (6) developed the point in even more foundational terms. What is a precise normal reference population in large-scale datasets explored at increased granularity? With more patient features or attributes retrieved from data, superior chances exist that normality definition can change by adapting to novel feature combinations.

Another challenge is estimating treatment effects, something which differs from predicting outcomes. The common population average effect analysis needs to be compared with heterogeneous effect analysis, this latter reflecting stratified medicine aimed at correcting for variability (4). Once again, with more observed characteristics more patients can be differentiated in relation with response to treatment, although in decreasing sample sizes. Taken to an extreme, personalized effect analysis can be targeted to single-patient approaches based on multiple criteria to evaluate treatment options and thus weighting many variables in support of decisions, including preference data [Salmasi and Capobianco (27) on the use in EHR-driven models of instrumental variables for estimation of person-centered treatment effects (PeT), following (28)].

Finally, the issue of interoperability and the difficulty of modeling it. Interoperability refers usually to systems exchanging information, and it is necessary to know when systems can be interoperable and what they can exchange (29). However, another interconnected aspect is model interoperability, i.e., how to align model structures such that the same reference (say a model family) can be used to assess their validity and similar data can be used for learning to generalization tasks. A possible strategy is to identify elements that the models have in common and to combine them into a so-called core or backbone model. Unlike ensemble models that operate an aggregation of predictions from a variety of models to increase prediction accuracy and/or reduce overfitting effects, this core strategy enables a synthesis of separate characteristics that models share to be leveraged for inference scopes. This methodological strategy ensures the possibility of preserving communication between such key model characteristics and allows an expansion of the possible outreach across multiple domains. In the presence of limited throughput and scalability issues, the core model performance could be enhanced by sharding, an approach typical of databases (and recently blockchains) designed to partition large datasets into smaller ones (shards) in order to increase the overall system's efficiency.

## Author Contributions

The author confirms being the sole contributor of this work and has approved it for publication.

### Conflict of Interest

The author declares that the research was conducted in the absence of any commercial or financial relationships that could be construed as a potential conflict of interest.
